# RETRACTION: The Effect of Liposomal Bupivacaine for Surgical Wound Infiltration: A Meta‐Analysis of Randomised Controlled Trials

**DOI:** 10.1111/iwj.70563

**Published:** 2025-04-18

## Abstract

**RETRACTION:** JiangH. , MaQ. , DongJ. , and YeX. , “The Effect of Liposomal Bupivacaine for Surgical Wound Infiltration: A Meta‐Analysis of Randomised Controlled Trials,” International Wound Journal 20, no. 5 (2023): 1591–1608, 10.1111/iwj.14015.36345868 PMC10088822

The above article, published online on 08 November 2022, in Wiley Online Library (http://onlinelibrary.wiley.com/), has been retracted by agreement between the journal Editor in Chief, Professor Keith Harding; and John Wiley & Sons Ltd. Following an investigation by the publisher, all parties have concluded that this article was accepted solely on the basis of a compromised peer review process. The editors have therefore decided to retract the article. The authors did not respond to our notice regarding the retraction.

**RETRACTION:** H. Jiang , Q. Ma , J. Dong , and X. Ye , “The Effect of Liposomal Bupivacaine for Surgical Wound Infiltration: A Meta‐Analysis of Randomised Controlled Trials,” International Wound Journal 20, no. 5 (2023): 1591–1608, 10.1111/iwj.14015.36345868 PMC10088822

The above article, published online on 08 November 2022, in Wiley Online Library (http://onlinelibrary.wiley.com/), has been retracted by agreement between the journal Editor in Chief, Professor Keith Harding; and John Wiley & Sons Ltd. Following an investigation by the publisher, all parties have concluded that this article was accepted solely on the basis of a compromised peer review process. The editors have therefore decided to retract the article. The authors did not respond to our notice regarding the retraction.

